# Models, frameworks and theories in the implementation of programs targeted to reduce formal coercion in mental health settings: a systematic review

**DOI:** 10.3389/fpsyt.2023.1158145

**Published:** 2023-06-15

**Authors:** Tella Lantta, Joy Duxbury, Alina Haines-Delmont, Anna Björkdahl, Tonje Lossius Husum, Jakub Lickiewicz, Athanassios Douzenis, Elaine Craig, Katie Goodall, Christina Bora, Rachel Whyte, Richard Whittington

**Affiliations:** ^1^Department of Nursing Science, University of Turku, Turku, Finland; ^2^Department of Nursing, Manchester Metropolitan University, Manchester, United Kingdom; ^3^Centre for Psychiatric Research, Department of Clinical Neuroscience, Karolinska Institutet, Stockholm Health Care Services, Stockholm, Sweden; ^4^Department of Health Sciences, Oslo Metropolitan University, Oslo, Norway; ^5^Centre for Medical Ethics, University of Oslo, Oslo, Norway; ^6^Department of Health Psychology, Jagiellonian University Medical College, Krakow, Poland; ^7^Second Psychiatry Department, Attikon University Hospital, National and Kapodistrian University of Athens, Athens, Greece; ^8^Second Psychiatry Department, Attikon University Hospital, National and Kapodistrian University of Athens Medical School, Athens, Greece; ^9^Centre for Research and Education in Security, Prisons and Forensic Psychiatry, Forensic Department Østmarka, St. Olav's Hospital, Trondheim, Norway; ^10^Department of Mental Health, Norwegian University of Science and Technology (NTNU), Trondheim, Norway; ^11^Department of Public Health, Policy and Systems, University of Liverpool, Liverpool, United Kingdom

**Keywords:** implementation science, mental health, psychiatric care, coercive measures, coercion, intervention, implementation tools

## Abstract

**Introduction:**

Implementation models, frameworks and theories (hereafter tools) provide researchers and clinicians with an approach to understand the processes and mechanisms for the successful implementation of healthcare innovations. Previous research in mental health settings has revealed, that the implementation of coercion reduction programs presents a number of challenges. However, there is a lack of systematized knowledge of whether the advantages of implementation science have been utilized in this field of research. This systematic review aims to gain a better understanding of which tools have been used by studies when implementing programs aiming to reduce formal coercion in mental health settings, and what implementation outcomes they have reported.

**Methods:**

A systematic search was conducted using PubMed, CINAHL, PsycINFO, Cochrane, Scopus, and Web of Science. A manual search was used to supplement database searches. Quality appraisal of included studies was undertaken using MMAT—Mixed Methods Appraisal Tool. A descriptive and narrative synthesis was formed based on extracted data. Preferred Reporting Items for Systematic Review and Meta-Analyses (PRISMA) guidelines were followed in this review.

**Results:**

We identified 5,295 references after duplicates were removed. Four additional references were found with a manual search. In total eight studies reported in nine papers were included in the review. Coercion reduction programs that were implemented included those that were holistic, and/or used professional judgement, staff training and sensory modulation interventions. Eight different implementation tools were identified from the included studies. None of them reported all eight implementation outcomes sought from the papers. The most frequently reported outcomes were acceptability (4/8 studies) and adaptation (3/8). With regards to implementation costs, no data were provided by any of the studies. The quality of the studies was assessed to be overall quite low.

**Discussion:**

Systematic implementation tools are seldom used when efforts are being made to embed interventions to reduce coercive measures in routine mental health care. More high-quality studies are needed in the research area that also involves perspectives of service users and carers. In addition, based on our review, it is unclear what the costs and resources are needed to implement complex interventions with the guidance of an implementation tool.

**Systematic review registration:**

[Prospero], identifier [CRD42021284959].

## 1. Introduction

### 1.1. Implementation theories, models, and frameworks

Implementation science has its origins in the 1990's with the rise of evidence-based practice in the field of medicine. This evidence-based movement noted that research findings and empirically supported practices should be more widely spread and applied to achieve improved health and welfare for populations. However, it was evident that the implementation of these effective practices and findings was facing many challenges. Thus, it was assumed that research into implementation itself as a process can create knowledge to close or reduce the gap between evidence and practice (1).

Implementation theories, models and frameworks are three different types of conceptual tool which provide insights into the mechanisms by which implementation is more likely to succeed (2). Implementation theories are generally specific and predictive. They propose directional relationships between concepts making them suitable for hypothesis testing as they may guide what may or may not work (3). Models are often more specific and prescriptive: for example, describing steps in the implementation process. They are commonly used to describe and guide the process of translating research into practice (2). Frameworks in contrast usually organize, explain or describe information and relationships between concepts (4). A framework gives a “structure, overview, outline, system or plan consisting of various descriptive categories,” as stated by Nilsen (2). As opposite to theories, models or frameworks do not specify the mechanisms of change, but are more like checklists of factors relevant to various aspects of implementation (2). Each of these constructs will have one of the following aims: (1) process models that describe or guide the implementation process, (2) determinants frameworks, classic theories, and implementation theories that aim to understand/or explain what influences implementation outcomes, and (3) evaluation frameworks evaluating or measuring the success of implementation (5).

### 1.2. Implementation of coercion reduction programs in mental health settings

In mental health settings, coercion can be defined as forceful action, involuntary treatment, or threats undertaken in the course of providing treatment or addressing perceived harm that a person poses to themselves or others (6). Examples of formal coercion include mechanical restraint using belts, manual restraint, seclusion or physically enforced administration of medication. The use of coercion has multiple known negative effects on service users, including psychological (7) and physical harm and even death (8).

Reducing or ending the use of coercion is one of the key health policy issues in mental health services worldwide (6, 9). Although having shared goals, the use of coercion has a great amount of variation between regions and countries. Sources of this variation include different service configurations, different mental health laws, and different social policies and cultures (10).

Many successful programs have been developed and tested to reduce coercion (9). However, there are issues in implementing these programs in mental health settings, as has been noted with other evidence-based practices in mental health and beyond (1). A recent European survey in 17 countries showed that two forms of coercion, seclusion and physical restraint, were still the most used techniques to manage service users' aggression in mental health settings, but variations between countries exist also here (11). So, the successful coercion-reduction programs do not seem to be adopted into current practice despite good evidence of their efficacy. This is a clear example of an implementation problem, which might benefit from an implementation science approach, i.e., that clinicians do not implement these programs at all, or if they do, these programs do not have their intended effect (efficacy-effectiveness gap) and/or the clinicians do not accept the implementation outcomes. With new coercion-reduction programs, it might be that clinicians are not engaged with using the new practice (12, 13), they are not accepting the intervention and their negative attitudes have an impact on how the program is implemented and sustained (13). Other potential reasons might be that the intervention is too difficult to use (14) and the environment for the implementation has high acuity and therefore there is not enough time or resources for adopting new interventions (13).

Previous systematic reviews on this topic have focused on the question of effectiveness of coercion reduction programs (15) rather than implementation issues, giving an overview of existing interventions (16, 17), or focusing on single programs, such as Safewards (13). There have been systematic implementation reviews in mental health services such as one that looked at effective strategies when implementing trauma-informed care in youth inpatient psychiatric and residential treatment settings (18). But as far as we are aware, there have not been any systematic attempts to review implementation theories, models and frameworks in the implementation of coercion reduction programs in mental health settings. Lack of awareness of the extent to which implementation of coercion reduction programs has utilized implementation science prevents understanding fully the obstacles to be overcome when translating evidence to practice.

The main aim of this systematic review therefore is to gain a better understanding of which models, theories and frameworks (hereafter all referred to as tools) have been used by studies when attempting to implement coercion reduction programs in mental health settings, and what implementation outcomes they have reported. We see this as an important step in growing understanding of the role of implementation science in coercion reduction and indicating future directions in research and practice in this area. This work is part of COST Action FOSTREN: Fostering and Strengthening Approaches to Reducing Coercion in European Mental Health Services (CA19133).

## 2. Materials and methods

The review was performed according to the Preferred Reporting Items for Systematic Reviews and Meta-Analyses (PRISMA) (19) guidance. The protocol is registered with Prospero (CRD42021284959).

### 2.1. Search strategy

The literature search was carried out from November 19 to 20, 2021, using the following databases: PubMed, CINAHL, PsycINFO, Cochrane, Scopus, and Web of Science. The search strategy was externally validated by a librarian. We also searched Google Scholar and references from included articles for additional studies. No restriction was used in the databases. The full search strategy for Web of Science is available as Supplementary material. We contacted the authors if a full-text version was not available.

We included implementation studies (any design) reporting on the implementation of non-pharmacological interventions in any of the mental health settings. We defined interventions as any new intervention or practice improvement effort related to patient care. We required interventions to be focused on aiming to reduce patient coercion and restrictions in care within an explicit implementation science framework. We defined formal coercion as including at least one of the following measures: seclusion, segregation, physical restraint, mechanical restraint, involuntary medication treatment, constant observation, intermittent observation, time out, net bed, open area seclusion, involuntary admission and care, and outpatient commitment, or restrictions in care as defined as coercion by individual studies. We required that included studies reported a referenced tool to guide, analyze or evaluate the implementation. We considered studies published in peer-reviewed journals, in any language and any year.

We excluded studies using mainly pharmacological treatments (drug studies), as these interventions may involve formal or informal coercion. Studies conducted outside healthcare settings, for example, schools or non-governmental organizations were excluded. We also excluded those studies where the implementation only consisted of a single strategy; and/or a specific tool had not been described. We did not consider letters, opinions, editorials, books, theses, study protocols, systematic reviews, or meta-analyses. The detailed PICO criteria were stated in the protocol as follows:

P (Participants, population) = The setting needed to be inpatient or outpatient care in the field of mental health care. No restrictions of age, diagnosis, or professional group were applied. We included both patients and professionals as participants.

I (Intervention) = The intervention was required to have two components. First, we considered studies utilizing any named and referenced implementation tool. Tools were required to have at least one of the three specific aims proposed by Nilsen (2): (1) describing and/or guiding the process of translating research into practice, (2) understanding and/or explaining what influences implementation outcomes and (3) evaluating implementation. They needed to have a detailed structure described in the included paper or in a cited reference. Second, the intervention being implemented had to be a non-pharmacological approach/technique used in a mental health setting. This included any new intervention or practice improvement effort related to patient care or education of professionals and coercion reduction programs.

C (Comparator, control) = We did not restrict comparators as the review did not focus solely on randomized controlled trials.

O (Outcomes) = We considered the following implementation outcome domains: acceptability, adaptation, appropriateness, costs, feasibility, fidelity, penetration, and sustainability (20), as defined by each study. Outcome domains were our primary outcome, irrespective of the differences among tools considered by the studies.

The titles and abstracts (Stage 1) and full text (Stage 2) were independently screened by two reviewers (TL, AH-D, AD, TLH, JD, CB, EC, JL, AB, and KG). Any disagreements/conflicts were resolved by a third reviewer (TL, AH-D, AB, and JD) who was not authoring the paper to be evaluated.

### 2.2. Data extraction

Data on implementation processes and other study characteristics were extracted from the included studies independently by two members of the research team (RiW and KG) and then reviewed by a third member (TL). Any disagreements and unclear items were discussed by RiW and KG, and a consensus was sought by validating the decision with TL.

Eight key implementation processes specified by Proctor et al. (20) were searched for in each of the studies. These processes are: acceptability, adoption, appropriateness, feasibility, fidelity, implementation costs, penetration and sustainability. Although these terms are clearly defined by Proctor et al. (20), their meaning as used in the literature is not fixed with much variability in how researchers employ each term.

Therefore, each study was initially examined using automated searching of truncated terms: accept^*^, adopt^*^, appropriate^*^, feasib^*^, fidelity^*^, cost^*^ (only when adjacent to “implementation”), penetrat^*^, and sustain^*^. This search was restricted to the empirical sections of each paper (i.e., methods and results).

If a term was detected in this way the following additional aspects were extracted:

1. Are data reported for this term? (yes, quantitative data only; yes, qualitative data only; yes, both types of data; no).
2. What types of respondents provided the data? (staff; patients; both; other).
3. Brief details of the reported data.

In addition, each study was examined with regard to whether its approach or method was based on a specified implementation checklist or tool. If so, the checklist name was extracted and the key implementation process from the eight specified above was noted (including an option for “multiple” processes).

Data on the study characteristics were also extracted for contextual purposes.

Both implementation outcomes and study characteristics coding sheets were piloted by completing the extraction forms on two studies. These were then discussed before agreeing to complete data extraction for all included papers. In addition, data extraction forms were cross referenced and double checked once completed. Any conflicts were discussed and resolved between the two people completing data extraction and a consensus was reached on all of these conflicts.

### 2.3. Study quality

Quality appraisal of included studies was undertaken using MMAT—Mixed Methods Appraisal Tool (21). MMAT is designed for systematic reviews that include qualitative, quantitative and mixed methods studies. It enables appraisal of the methodological quality of five categories of studies: qualitative research, randomized controlled trials, non-randomized studies, quantitative descriptive studies, and mixed methods studies. Scoring guidance provided by the developers of MMAT was followed (21). Appraisal was done by two authors (RaW and CB) and reviewed by a third (TL).

### 2.4. Data synthesis and analysis

The extracted data were first descriptively summarized using numbers, dichotomous yes/no categories, and text, as appropriate, followed by a narrative synthesis. We did not identify any relevant studies using randomized controlled study design and therefore were not able to include meta-analysis about effectiveness of the interventions to our study.

### 2.5. Amendments to information provided at registration

Because of the heterogeneity and small number of the studies, we were not able to report secondary outcomes of the studies (effectiveness) but focused only on the implementation outcomes. Study heterogeneity led us to develop a data extraction sheet that could capture all the important aspects of the included studies, instead of using JBI data extraction tools that do not include an implementation-specific extraction tool.

## 3. Results

### 3.1. Characteristics of included papers

**Figure 1:**
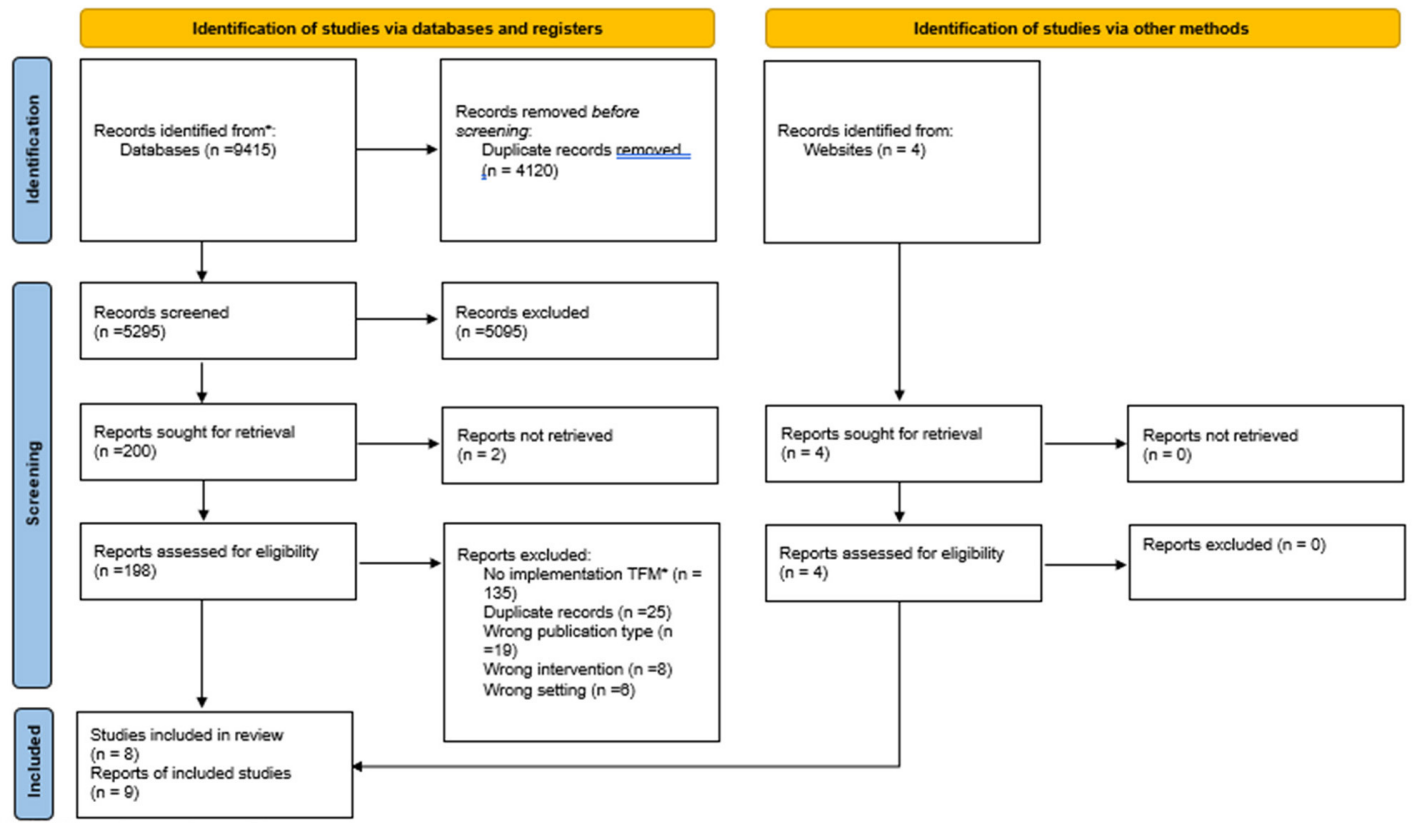
PRISMA 2020 flow diagram for new systematic reviews which included searches of databases, registers, and other sources. *TFM, Theory, framework or model. Adapted from Page et al. (19) with permission under the Creative Commons CC BY 4.0 license. For more information, visit http://www.prisma-statement.org/.

Our search generated 5,295 references after duplicates were removed. Four additional references were found with a manual search. After application of the inclusion and exclusion criteria eight studies reported in nine papers were included in the review (see PRISMA flow diagram Figure 1). Papers excluded at full-text screening (*N* = 193, including 24 duplicates, *n* = 169) are listed in Supplementary material 2.

Studies were conducted in Australia (*n* = 3, 37.5%), the USA (*n* = 2, 25.0%), Finland, the Netherlands and Germany (all *n* = 1, 12.5%). They were all written in English language. Implementation was most commonly studied within a non-randomized (22–24) or mixed-method study design (25–28). All studies were conducted in inpatient settings, either in forensic mental health (23) or general psychiatric wards (22, 24, 28–30), or in mixed settings, including both forensic and general mental health wards (25–27). Implementation periods (an active time that an intervention was implemented, as defined by each study) varied between 1 and 17 months, and in one study the length of the period was not stated (30).

### 3.2. Quality appraisal

Table 1 summarizes the quality appraisal of the included studies against MMAT criteria. Two of the qualitative studies (29, 30) fulfilled all the criteria set (7/7). For the quantitative and mixed-methods studies, many items could not be rated because insufficient information was provided in the papers.

**Table 1:** Quality assessment using MMAT tool.

**References**	**Screening questions**		**Qualitive studies**					**Non-randomized studies**					**Quantitative descriptive studies**					**Mixed-methods studies**					**Total points**
	S1	S2	1.1	1.2	1.3	1.4	1.5	3.1	3.2	3.3	3.4	3.5	4.1	4.2	4.3	4.4	4.5	5.1	5.2	5.3	5.4	5.5	
Baumgardt et al. (22)	1	1	N/A	N/A	N/A	N/A	N/A	1	1	1	0	0	N/A	N/A	N/A	N/A	N/A	N/A	N/A	N/A	N/A	N/A	5/7
De Beuf et al. (23)	1	1	N/A	N/A	N/A	N/A	N/A	0	1	1	1	1	N/A	N/A	N/A	N/A	N/A	N/A	N/A	N/A	N/A	N/A	5/7
Fletcher et al. (25)	1	1	1	1	1	1	1	N/A	N/A	N/A	N/A	N/A	1	-	1	-	-	1	1	1	1	1	14/17
Hale and Wendler (24)	1	1	N/A	N/A	N/A	N/A	N/A	-	-	0	-	-	N/A	N/A	N/A	N/A	N/A	N/A	N/A	N/A	N/A	N/A	2/7
Higgins et al. (29)	1	1	1	1	1	1	1	N/A	N/A	N/A	N/A	N/A	N/A	N/A	N/A	N/A	N/A	N/A	N/A	N/A	N/A	N/A	7/7
Lantta et al. (26, 27)	1	1	1	1	1	0	1	N/A	N/A	N/A	N/A	N/A	1	1	1	-	1	1	1	1	1	1	15/17
Repique et al. (28)	1	1	1	1	1	0	1	0	1	0	0	1	N/A	N/A	N/A	N/A	N/A	0	0	0	1	0	9/17
Wright et al. (30)	1	1	1	1	1	1	1	N/A	N/A	N/A	N/A	N/A	N/A	N/A	N/A	N/A	N/A	N/A	N/A	N/A	N/A	N/A	7/7

MMAT items: 1 = Yes; 0 = No; N/A =Not applicable in this study; - = Can't tell based on the article; S1 = Are there clear research questions? S2 = Do the collected data allow to address the research questions? 1.1 = Is the qualitative approach appropriate to answer the research question? 1.2 = Are the qualitative data collection methods adequate to address the research question? 1.3 = Are the findings adequately derived from the data? 1.4 = Is the interpretation of results sufficiently substantiated by data? 1.5 = Is there coherence between qualitative data sources, collection, analysis and interpretation? 3.1 = Are the participants representative of the target population? 3.2 = Are measurements appropriate regarding both the outcome and intervention (or exposure)? 3.3 = Are there complete outcome data? 3.4 = Are the confounders accounted for in the design and analysis? 3.5 = During the study period, is the intervention administered (or exposure occurred) as intended? 4.1 = Is the sampling strategy relevant to address the research question? 4.2 = Is the sample representative of the target population? 4.3 = Are the measurements appropriate? 4.4 = Is the risk of nonresponse bias low? 4.5 = Is the statistical analysis appropriate to answer the research question? 5.1 = Is there an adequate rationale for using a mixed methods design to address the research question? 5.2 = Are the different components of the study effectively integrated to answer the research question? 5.3 = Are the outputs of the integration of qualitative and quantitative components adequately interpreted? 5.4 = Are divergences and inconsistencies between quantitative and qualitative results adequately addressed? 5.5. Do the different components of the study adhere to the quality criteria of each tradition of the methods involved?

### 3.3. Implementation tools used by the studies

Eight different tools were identified from the included studies: none of them used the same approach Three of them were used to guide the implementation process, and the rest of them to evaluate or analyze the process (Table 2). We also identified additional checklists and tools that were used by the included studies to collect data related to implementation outcomes. Additional checklists and tools identified measured fidelity and included the Organization Fidelity Checklist and Safewards Fidelity Checklist.

**Table 2:** Implementation models, frameworks, or theories used guide, evaluate or analyze implementation processes.

	**To guide**			**To evaluate or analyze**				
**References**	**OMRU**	**Iowa model**	**Skolarus**	**IOF**	**CFIR**	**TDF**	**Behavioral**	**PARIHS**
			**and sales**				**change wheel**	
Baumgardt et al. (22)			x					
De Beuf et al. (23)				x				
Fletcher et al. (25)					x			
Hale and Wendler (24)		x						
Higgins et al. (29)							x	
Lantta et al. (26, 27)	x							
Repique et al. (28)								x
Wright et al. (30)						X		

Note. OMRU, Ottawa Model of Research Use; Iowa Model, Iowa Model for Evidence Based Practice-Revised; Skolarus & Sales, Skolarus & Sales implementation approach; IOF, Implementation Outcomes Framework; CFIR, Consolidated Framework for Implementation Research; TDF, Theoretical Domains Framework; PARISH, Promoting Action on Research Implementation in Health Services.

### 3.4. The interventions applied by the studies

We identified four types of interventions: holistic, professional judgement, staff training and sensory modulation. With regards to holistic, we classified Safewards (22, 25, 29) and Trauma-informed Care approaches (24). For professional judgement, we identified violence risk assessment either using the DASA (26, 27) or START:AV (23). The only staff training was a Recovery-Oriented Training Program (28) and one study used a sensory modulation approaches (30).

### 3.5. The implementation outcomes of the studies

We sought implementation outcomes as defined by Proctor et al. (20) from the eight included studies. A summary of the implementation outcomes is described in Table 3. None of the studies reported all of eight implementation outcomes. The number of implementation outcomes mentioned varied between 3 and 5 outcomes. Acceptability (seven out of nine papers), appropriateness (8/9) and sustainability (7/9) were most commonly named in the papers, whereas penetration was found in only one of the studies. However, most of the studies only mentioned an outcome by the name in their paper and did not report any actual data about the outcomes.

**Table 3:** Summary of the implementation outcomes.

**References**	**Acceptability**	**Adoption**	**Appropriateness**	**Feasibility**	**Fidelity**	**Implementation costs**	**Penetration**	**Sustainability**	Total^**^
Baumgardt et al. (22)	Yes	No	Yes	No	Yes	No	No	Yes	3/8
De Beuf et al. (23)	Yes	Yes	yes	Yes	No^*^	No	Yes	No	5/8
Fletcher et al. (25)	Yes	No	Yes	No	Yes	No	No	Yes	4/8
Hale and Wendler (24)	Yes	No	Yes	No	No	Yes	No	Yes	4/8
Higgins et al. (29)	Yes	No	No	Yes	Yes	No	No	Yes	4/8
Lantta et al. (26, 27)	Yes	Yes	Yes	Yes	No	Yes	No	No	5/8
Repique et al. (28)	Yes	Yes	No	No	No	Yes	No	Yes	4/8
Wright et al. (30)	Yes	No	Yes	No	No	No	No	Yes	3/8
Total^***^	7/9	4/9	8/9	3/9	4/9	3/9	1/9	7/9	-
Total with data^****^	4/9	3/9	2/9	2/9	2/9	0/9	1/9	1/9	-

^*^Reported in a separate paper, unreferenced.

^**^Total number of implementation outcomes found from the paper.

^***^Total number of studies mentioning the outcome.

^****^Total number of studies reporting data about the outcome.

In the next section, we will report a narrative, outcome by outcome, based on the data found.

#### 3.5.1. Acceptability

Acceptability is the perception among implementation stakeholders that a given treatment, service, practice, or innovation is agreeable, palatable, or satisfactory (20). Four papers reported data about acceptability (22, 23, 27, 30).

In most of these studies (22, 23, 30), acceptability of the intervention was evaluated from the staff's viewpoint with mixed views expressed toward the intervention. Baumgardt et al. (22) evaluated acceptability of Safewards by staff before the implementation period began. Staff who were not willing to use the intervention were given the option to change their working unit. Two out of 40 used this possibility. Staff in the study by De Beuf et al. (23), were increasingly dissatisfied over time (100% of the staff in time point two) with the implemented violence risk assessment tool START:AV, although they did find that the content of the tool was acceptable and not too complex. However, they were unconvinced of the credibility of the intervention i.e., they did not believe that START:AV would help them to prevent violent events. Staff in the study by Wright et al. (30) thought that the intervention, sensory modulation, was acceptable for some patients, but too risky to be used for highly distressed patients.

In contrast, Lantta et al. (27) evaluated the acceptability of the DASA violence risk assessment tool from the patient's perspective. They found that the acceptability of the tool and the research process, measured by patient's willingness to give written informed consent for the study, varied between wards where the implementation took place but remained low as a whole (17% of the patients).

#### 3.5.2. Adoption

Adoption is defined as the intention, initial decision, or action to try or employ a program (20). Three papers reported data about adoption (23, 27, 28) all from the staff's perspectives.

All three studies found that there is scope for improving adoption of the intervention during and after the implementation period. De Beuf et al. (23) evaluated adoption of the intervention by measuring how frequently the START:AV tool was used to assess patients' violence risk. They found that the percentage increased slowly over time, from 74 to 78%, but did not reach the set goal of an 80% completion rate. Completion rate also varied between individual assessors (range 29–100%). Lantta et al. (27) reported similar findings with another violence risk tool, the DASA. In their study, a 64% completion rate was reached, but it varied substantially between wards involved in the implementation ranging between 15 and 89%.

Repique et al. (28) used staff focus groups to evaluate adoption of recovery-oriented principles in care after a training program. According to their findings, staff were doing a “decent job” [sic] with incorporating recovery principles. However, they felt that more buy-in was still needed among staff, and it should start from the leadership level.

#### 3.5.3. Appropriateness

Appropriateness is the perceived fit, relevance, or compatibility of the program for a given practice setting, clinician or service user; and/or perceived fit to address a particular issue or problem (20). Two papers reported data about appropriateness (23, 30) and their respondents were staff members in both cases.

These two studies evaluated appropriateness from a slightly different viewpoint. In the De Beuf et al.'s (23) study staff evaluated if the intervention was useful for treatment. Again, there were diverse views. After the implementation period, 33% disagreed that the START:AV was useful. Still, there was a substantial proportion of staff who agreed with its usefulness as a whole (67%) and complete agreement of its usefulness concerning different factors of the tool (100%). In the Wright et al.'s (30) study, focus groups with staff revealed both appropriate and inappropriate ways sensory modulation approaches had been used in care, For example, a sensory room had been used as a place to play video games, not as a calming area for patients.

#### 3.5.4. Feasibility

Feasibility is defined as the extent to which a new program can be successfully used or carried out within a given setting (20). Two papers reported data about feasibility from the staff viewpoint (23, 27).

De Beuf et al. (23) asked staff about their intervention's practicality. Staff thought that they lacked time to use the intervention. They also reported that it took much longer to complete the START:AV assessment than indicated from previous studies. Lantta et al. (27) evaluated how the intervention actually worked. They evaluated how well the DASA predicted aggression in the wards. That outcome reached the set goal (AUC ≥ 0.70), varying between different forms of aggression 0.75–0.93.

#### 3.5.5. Fidelity

Fidelity is defined as the degree to which a program was implemented as it was planned in the original protocol or as it was intended by the program developers (20). Two of the included studies provided data on fidelity (22, 25) based on staff implementation activities.

Both studies reported a high level of fidelity when implementing Safewards. Baumgardt et al. (22) reported that wards were able to fully implement eight out of the 10 Safewards interventions, indicating high fidelity. Fletcher et al. (25) also had high implementers among their participating wards (8–10 interventions out of 10), but also some wards were only implementing 1–4 interventions.

#### 3.5.6. Implementation costs

Cost is defined as the cost impact of an implementation effort (20). None of the included studies provided data about implementation costs.

#### 3.5.7. Penetration

Penetration is defined as the integration of a program within a service setting and its subsystems (20). De Beuf et al. (23) was the only paper providing information on this outcome. They asked the staff if the intervention (START:AV) was integrated in the setting's treatment plans and case conferences. First, a negative result of 100% of staff disagreeing was reported on the question if the tool contributed to more effective communication and whether it increased structure during case conferences. This result did not change over time. Despite this negative finding, the integration of the tool into the treatment process seemed to improve over time based on the second question evaluating penetration. The proportion of staff who disagreed that the tool was sufficiently integrated decreased from 75 to 17%.

#### 3.5.8. Sustainability

Sustainability is defined as the extent to which a newly implemented program is maintained or institutionalized within a service setting's ongoing, stable operations (20). Only Hale and Wendler (24) reported data for the sustainability of the intervention and this was from the staff viewpoint. According to their results, there was a 9.3% reduction of physical holding and seclusion 12 months later after implementing trauma-informed care in children and adolescent inpatient services.

## 4. Discussion

Over the past decade, several promising coercion reduction programs that constitute complex interventions in mental health settings, have been developed and reported to be successful (9).

However, the use and testing of implementation tools, based on the principles of implementation science, in this context appear in our review to be in its infancy. We screened 204 full-text versions of coercion reduction intervention studies but of those we could only find nine (4.4%) that had used a named implementation tool. This indicates that although there are many reportedly successful reduction studies published, the extent and quality of the implementation of the intervention and the sustainability over time is often unknown. Consequently, there is a lack of knowledge about which coercion reduction programs are robust enough to be successful under routine less-than-ideal conditions in clinical settings where interventions may be only partially or poorly implemented.

Using the MMAT scoring system, we found that the quality of the included studies was mostly quite low, with the exception of two qualitative papers (29, 30). The implementation process was generally poorly reported with significant variation in what was reported and how. Tools were mostly used to evaluate or analyze the implementation, not to guide it. Similarly, all studies did mention three to five of the implementation outcomes we used but half of the studies provided no data on them. To make future replications and comparisons between different implementation approaches possible, there is a need to find a more standardized and streamlined process for reporting of specific implementation aspects when introducing coercion reduction programs into mental health settings. This is an acknowledged need in general with implementation studies in a wide range of applied settings, not only in this field of research (31).

There may be several reasons for the low prevalence of implementation tools used in coercion reduction studies. Greenhalgh argues that real-world challenges to implementation of evidence-based practice are often characterized by the uniqueness, complexity and incomplete, contradictory and changing requirements of identified barriers (32). Further, Greenhalgh states that because of the messiness of the real-world context, there will never be a perfectly fitted tool to choose. Instead, theoretical tools should be approached carefully but pragmatically, without any expectation of finding a tool that will completely solve the implementation issues. Rather the tool should be seen more modestly as a way of organizing thoughts and ideas about complex challenges.

At the same time, the approach suggested by Greenhalgh would make it difficult to compare the effectiveness of different implementation strategies across studies to enhance the effectiveness of coercion reduction programs if they were all pragmatically set up based on each clinical setting's own local “messiness.” We therefore believe there is a need for some standardized reporting of implementation outcomes. Possibly, a way forward could be a template for reporting key elements of an implementation study, based on an overarching generic terminology. If not too highly detailed, this would cover the use of different implementation tools and still enable useful comparisons to be made. To meet this need, Pinnock et al. (31) developed the guideline and checklist StaRI (Standards for Reporting Implementation Studies Statement). StaRI includes the parallel reporting of both the implementation strategy, regardless of implementation tool used, and the effectiveness of the intervention implemented. To enhance the quality of future intervention studies on coercion reduction, StaRI could be one option to address the challenges of structured reporting on both the intervention effectiveness and the implementation strategy.

Each of the nine papers in this review used a different implementation tool (Table 2) which suggest that coercion reduction programs are at different stages of development or implementation. Only three of these studies described a somewhat clear rationale behind the choice of the tool. It is therefore unclear if the tools were chosen based on an assessment of which tool would be best suited or for some other reason. To provide clarity on what distinguishes different implementation models, and to aid the choice of a tool, Nilsen (5) has suggested five tool categories: process models, determinant frameworks, classic theories, implementation theories and evaluation frameworks. Of the nine papers here, two were process models (OMRU, Iowa model), three were determinant frameworks (CFIR, TDF, and PARIHS), one was an implementation theory (BCW), one was an evaluation framework (IOF), and one a tool by Skolarus and Sales. Most authors used their chosen tool to analyze and evaluate the implementation process retrospectively, a purpose that might not be best suited for process models which aim at describing and guiding the process of translating research into practice (1).

In our review we included only overarching implementation tools and excluded studies that referred only to practices based on improvement science, such as the PDSA (plan-do-study-act)-cycle. It is possible that improvement strategies which tend to focus on very specific ways of introducing interventions, so “how” to implement new practice (quality improvement work) are more commonly used in intervention studies than the more theoretically driven implementation tools that focus on “what” should be included in implementation strategies. At the same time, Leeman et al. (33) argue that it is time that the fields of implementation and improvement science should start to align. An alignment would, according to the authors, benefit both areas of knowledge by reducing the research/practice gap, fostering local ownership of implementation, generating evidence, developing context-specific implementation strategies and building practice-based evidence capacity to improve care (33).

The studies in this review evaluated between three and five components out of the eight we extracted. With limited resources in clinical settings, it is not known whether outcome measurement quality improves when multiple components are used. It is more likely that this quality declines as the implementation strategy becomes more complex and it may be more effective to focus on only one or two components with the capacity but to evaluate them really well. The effectiveness of the implementation strategy chosen could depend more on whether there was an a priori rationale for the strategy, based on an assessment of expected enablers and barriers, rather than the number of implementation strategy components (34). This thought is further strengthened by a review of systematic reviews on interventions to change health-care professionals' behaviors, by Squires et al. (35), which concluded that there was no evidence to suggest that multifaceted strategies were more effective than single-component strategies. This finding should be further explored for the relevance to coercion reduction studies as it could offer important guidance for clinical settings with limited resources (35).

Acceptability (4/9) and adoption (3/9) were the most popular components reported upon (Table 3). Appropriateness and acceptability are conceptually close terms that have been found to overlap and be used inconsistently in the literature. However, they are not synonyms, and a new intervention can be assessed as appropriate but not acceptable, due to, for example, cost (20). Similarly, appropriateness has been used interchangeably with terms like perceived fit, relevance, compatibility, suitability, usefulness, and practicability (36), highlighting the challenges involved regarding conceptual clarity when planning an implementation study and reviewing the literature. In order to continue to develop this field, it would be useful to find a common conceptual framework to promote implementation work in the future. This is in line with the recommendation by Proctor et al. (20).

Furthermore, given the resources needed in clinical settings to successfully implement a new complex intervention, sustainability could be viewed as one of the most valid measures of implementation itself. It can be objectively evaluated through behavioral observation and inherently indicates the ongoing adoption of an innovation over long time periods post-introduction. However, Wiltsey Stirman et al. (37) found in their implementation review of empirical literature including sustainability aspects of implementation, that <½ of the studies presented data on sustainability outcomes. It was also evident that what was considered a clinically meaningful sustainability measure varied between the studies so again consensus was a problem. Examples of different measures were the proportion of sites that implemented the intervention over time, the proportion of patients receiving the intervention, and the level of desired patient outcomes (37). Sustainability measures are therefore considered a challenge due to the unclear definitions of what should be measured and when to assess the level of sustainability (38).

Penetration (1/9), implementation costs (0/9) and sustainability (1/9) were the least used components in our review. According to Proctor et al. (20), penetration as a concept is not commonly used in the literature. Lack of penetration can be an indication that staff might be reluctant to change as found by De Beuf et al. (23). It is probably useful to identify and address resistance to change in organizations to enhance successful implementation of interventions, for example by use of ORIC (Organizational Readiness for Implementing Change) (39).

None of the identified studies provided data about implementation costs. This is in line with the findings of a recent review (40) about implementation of early psychosis services in Latin America where they too did not identify any studies reporting costs of implementation. At a time when there is more focus on the costs of healthcare services, this is also a possible area of improvement in implementation reporting. Economic implementation studies with standard economic costing methods are warranted in mental health areas (41).

To increase the use of implementation tools in coercion reduction projects, we believe the advantages gained from their contribution to implementation processes, such as clinically meaningful outcomes and more rigorous evaluations of what implementation strategy will enhance intervention effectiveness and efficacy, need to be clarified and demonstrated. Moreover, there is a need to identify measures for implementation outcomes that can monitor and evaluate implementation determinants, mechanisms, processes, strategies and outcomes (42). Implementation tools should be evidence based, and when operationalized in clinical research, the measurement tools associated with them should be statistically tested for psychometric properties, including the capacity to discriminate between different tool items (36). Promising psychometric research like this is ongoing. Studies have for example been conducted to develop and psychometrically test measures for acceptability, appropriateness and feasibility (42), as well as to assess the validity and reliability of seven domains of the CFIR implementation tool (43).

It is noteworthy that only one of the studies in this review (27) reported on any type of service user involvement or patient perspectives on implementation process or its evaluation. It could be argued that since staff are responsible for implementing interventions as part of their professional employment, it is only their point of view which is relevant. Interventions to be implemented in coercion reduction have also lacked user involvement (44), likewise research in the area (45). However, with increasing emphasis on formal collaboration in treatment and care, full implementation requires an understanding of how to engage service users in the process.

### 4.1. Limitations

A potential limitation of this review is linked to selection bias; it is possible that some articles were not identified at screening stage because of the different terminologies used. As highlighted above, the fuzziness of the core concepts made it hard to apply inclusion/exclusion criteria with confidence. The decision to use truncated terms in data extraction and not to include possible synonyms may have led us to overlook some of the results in the included papers. However, as the implementation outcomes were not fully defined in the papers, we were not able to include analogous synonyms in our analysis with a risk of over-interpreting the meaning of these terms. Overlapping terms and terminologies used to define or describe the same concepts pointed to the need to clarify language and definitions, although, as this review suggest, even an existing “umbrella tool” is not the best way forward, as too complex and not feasible to apply in practice.

One of the authors of this review (TL) was a lead author of two of the included studies. To avoid the potential conflict of interest when screening, the inclusion of the papers involving members of the review team was done by reviewers outside of the particular study. In addition, the main responsibility for the data extraction was in the hands of authors (RiW and KG) not involved in the included studies.

There is also the limitation due to underlying bias within the included studies, as these were only from so-called “Western Europe,” Australia and the United States, missing the cultural diversity, historical background and approaches to healthcare in other countries, especially African, Asian and Eastern European countries. A limitation inherent to any systematic review is that its quality relies heavily on the methodological rigor and biases of included papers. Our quality appraisal indicated that only 2/7 studies fulfilled all the MMAT criteria; many items could not be rated due to lack of insufficient information, especially in the quantitative/mixed methods papers.

Finally, due to the diversity of studies included—different tools, methodology, terminology, and the gaps in reporting, it was difficult to synthesize across and report on any robustly established mechanisms.

## 5. Conclusion

An impetus to minimize the use of coercion and its negative impact in mental health settings has been gaining momentum for some time now, globally. This has resulted in an increase in research in this area, policies to support this drive and practice-based initiatives to facilitate and support less restrictive environments and relationships. However, the implementation of such approaches has been hindered or at least poorly reported upon by the complexities of frameworks currently proposed, leading to lost opportunities for feasible, impactful and sustained interventions to be easily introduced into many practice settings.

From our review it is clear that systematic implementation tools appear to be seldom used when efforts are being made to embed interventions aimed at reducing use of coercive measures in routine mental health care. This is compounded by evidence from clinical experience that it can be difficult to implement an intervention in a new setting and that it is often not sustainable across time (46).

Most notably, the lack of clear descriptions about the underlying stages and principles needed to support the implementation of evidence in a meaningful way is lacking, as demonstrated in the small number of studies that we were able to report upon in this review. In particular matters relating to the costs and resources needed to implement complex interventions in settings where there are vulnerable populations is poorly considered. Additionally, the need to incorporate the views of all stakeholders in the “how” to successfully change practice for the better is crucial and the service user voice is missing.

To improve implementation efforts, the quality of mental health care services, and indeed to minimize the use of coercion, greater efforts are required to make the world of implementation science more accessible. There is a real need to identify and adequately describe the use of achievable, targeted and well-explained frameworks that allow change to be enacted upon and maintained. The use of streamlined, comprehensive, and less costly implementation tools should be more freely available, with the necessary workforce development in how to use and evaluate their impact in mental health care. Only then can we improve our practices and services in a way that is important to and valued by those using or working in care settings to allow for positive outcomes that can be replicated elsewhere.

## Data availability statement

The original contributions presented in the study are included in the article/Supplementary material, further inquiries can be directed to the corresponding author.

## Author contributions

TL, JD, AB, and RWhi: conceptualization. TL, JD, AH-D, AB, TLH, JL, EC, KG, CB, AD, RWhy, and RWhi: design, methodology, and conduction of the study. TL, RWhi, and KG: analysis and interpretation. TL: writing—original draft preparation. AB, TLH, JD, KG, EC, JL, AH-D, CB, RWhy, and RWhi: writing—review and editing. All authors contributed to the article and approved the submitted version.
