# Supplementary material for: Models, frameworks and theories in the implementation of programs targeted to reduce formal coercion in mental health settings: a systematic review
Source: Front Psychiatry. 2023 Jun 15;14:1158145. doi: 10.3389/fpsyt.2023.1158145 (PMC10311067; doi:10.3389/fpsyt.2023.1158145)
Supplement: Supplementary file 1 [file Data_Sheet_1.docx]

Supplementary Material

**Models, frameworks and theories in the implementation of coercion reduction programs in mental health settings: a systematic review**

**Tella Lantta*, Joy Duxbury, Alina Haines-Delmont, Anna Björkdahl, Tonje Lossius Husum, Jakub Lickiewicz, Athanassios Douzenis, Elaine Craig, Katie Goodall, Christina Bora, Rachel Whyte, Richard Whittington**

*** Correspondence:** Corresponding Author: [tella.lantta@utu.fi](mailto:tella.lantta@utu.fi)

# Supplementary Data

The full search strategy for the Web of Science, 19.11.2021

(implement* OR “knowledge transfer” OR diffusion OR disseminat* OR translat*) (Topic) and (strateg* OR intervention* OR program* OR plan* OR process* OR model* OR framework* OR theor*) (Topic) and ((psychiatric OR forensic OR "mental health" OR psycholog*) AND (nurs* OR care OR hospital* OR unit* OR ward* OR setting* OR institution*)) (Topic) and (coerci* OR containment* OR restrain* OR "restrictive intervention*" OR “restrictive practice*” OR isolat* OR seclusion* OR "forced medication" OR "constant observation" OR "intermittent observation" OR "open area seclusion" OR "time out" OR "forced injection" OR "net bed" OR "rapid tranq*" OR "enforced medication" OR "outpatient commitment" OR "involuntary care" OR "involuntary admission" OR "compulsory admission" OR segregation OR prone OR “clinical hold*” OR “physical intervention*” OR "blanket rule*" OR “blanket restriction*”) (Topic)

# Supplementary Table

| Authors, Year | Reference | Intervention |
| --- | --- | --- |
| Andersen et al. 2017 | Applying sensory modulation to mental health inpatient care to reduce seclusion and restraint: A case control study. | Sensory modulation |
| Anderson et al. 2021 | Influence of organizational climate and clinician morale on seclusion and physical restraint use in inpatient psychiatric units. | n/a |
| Andrassy et al. 2016 | Feelings Thermometer: An early intervention scale for seclusion/restraint reduction among children and adolescents in residential psychiatric care. | Feelings Thermometer -scale |
| Application 2012 | The Restraint Theory & the Construction and Implementation of One-stop Integrated Open Service Management Pattern | Integrated Open Service Management Pattern |
| Aremu et al. 2018 | Implementation of Trauma-Informed care and Brief Solution-Focused therapy a quality improvement project aimed at increasing engagement on an inpatient psychiatric unit. | Trauma-Informed Care andBrief Solution-Focused Therapy |
| Ashcraft et al. 2012 | The development and implementation of ‘no force first” as a best practice. | “No Force First” policy |
| Ashcraft et al. 2008 | Eliminating seclusion and restraint in recovery-oriented crisis. | Multiple interventions |
| Ayres et al. 2021 | Recovery Model Implementation for a Medical/Geriatric Psychiatric Unit to Decrease Restraint and SeclusionEpisodes: A Quality Improvement Project. | Recovery Model |
| Azeem et al. 2017 | Effectiveness of six core strategies based on trauma informed care in reducing seclusions and restraints at a child and adolescent psychiatric hospital. | Trauma Informed Care |
| Azeem et al. 2011 | Effectiveness of six core strategies based on trauma informed care in reducing seclusions and restraints at a child and adolescent psychiatric hospital. | Trauma Informed Care |
| Bak et al. 2018 | Changing attitudes to the use of coercion, through education. | Education in how to prevent physical coercive interventions. |
| Becker et al. 1997 | Intractability is relative: Behaviour therapy in the elimination of violence in psychotic forensic patients. | Integrated behaviour therapy program |
| Bell et al. 2016 | Succeeding in sustained reduction in the use of restraint using the improvement model. | Improvement Model (PDSA) |
| Bisconer et al. 2006 | Managing aggression in a psychiatric hospital using a behaviour plan: a case study. | Behaviour plan |
| Bell et al. 2016 | Succeeding in Sustained Reduction in the use of Restraint using the Improvement Model. | Improvement Model (PDSA) |
| Blair et al. 2017 | Reduction of seclusion and restraint in an inpatient psychiatric setting: A pilot study. | Intervention components included: the Brøset Violence Checklist, mandated staff education in crisis intervention and trauma informed treatment, formal administrative review of seclusion/restraint events and environmental enhancements. |
| Blair et al. 2015 | The engagement model for reducing seclusion and restraint: 13 years later. | The Engagement Model |
| Borckardt et al. 2011 | Systematic investigation of initiatives to reduce seclusion and restraint in a state psychiatric hospital. | Behavioral interventions: The Engagement Model, Trauma-Informed Care and the therapeutic environment intervention |
| Boumans et al. 2016 | ‘Kunnen de separatiecijfers omlaag?’ Structuur geven aan nehandelbeleid met methodisch werken. | n/a |
| Boumans et al. 2015 | Seclusion and the importance of contextual factors: An innovation project revisited. | An institutional program on coercive measures |
| Boumans et al. 2015 | The methodical work approach and the reduction in the use of seclusion: How did it work? | The Methodical Work Approach |
| Boumans et al. 2014 | Reduction in the use of seclusion by the methodical work approach. | The Methodical Work Approach |
| Bowers et al. 2008 | A replication study of the City nurse intervention: reducing conflict and containment on three acute psychiatric wards. | The City nurse intervention |
| Bowers et al. 2015 | Reducing conflict and containment rates on acute psychiatric wards: The Safewards cluster randomized controlled trial. | The Safewards Model |
| Bowers et al. 2014 | Safewards: the empirical basis of the model and a critical appraisal. | The Safewards Model |
| Brathovde 2021 | Improving the standard of care in the management of agitation in the acute psychiatric setting . | Best Practices in Evaluating and Treating Agitation (BETA), the Brøset Violence Checklist |
| Brooks et al. 2019 | Implementing an intervention designed to enhance service user involvement in mental health care planning: a qualitative process evaluation. | User- and carer-informed training package |
| Caldwell et al. 2014 | Successful seclusion and restraint prevention effort in child and adolescent programs. | Multiple interventions |
| Carlson et al. 2014 | Preventing restraint and seclusion: A multilevel grounded theory analysis. | n/a |
| Chang et al. 2014 | Effects of a recovery-oriented cognitive therapy training program on inpatient staff incidents of seclusion and restraint. | A recovery-oriented cognitive therapy (CT-R) milieu training program |
| Clark et al. 2020 | The use of positive behaviour support plans in mental health inpatient care: A mixed methods study. | Positive behaviour support Plans |
| Clavon et al. 1991 | Implementation of a restraint policy: A case study. | A no-restraint policy |
| Corrigan et al. 1995 | The effects of interactive staff training on staff programing and patient aggression in a psychiatric inpatient ward. | Interactive staff training |
| Craig et al. 2018 | Evaluation of a program model for minimizing restraint and seclusion. | Trauma Informed Approach model |
| Currier et al. 2002 | Use of restraint before and after implementation of the new HCFA rules. | Health Care Financing Administration(HCFA) rules on restraint and seclusion |
| Czernin et al. 2020 | Effects of the psychiatric care concept “Weddinger Modell” on mechanical coercive measures. | Weddinger Model |
| Davies et al. 2018 | Implementing positive behavioural support in a forensic psychiatric intensive care unit: addressing the barriers. | Positive behaviour support Plans |
| Davies et al. 2021 | An evaluation of the implementation of Safewards on an assessment and treatment unit for people with anintellectual disability. | The Safewards |
| Davis et al. 1985 | Reduction of patient violence without recourse to punishment. | Positive reinforcement privilege distribution system (PDS) |
| Dayton et al. 2021 | Restraint, seclusion, and implementation of positive behavior interventions and supports. | Positive behaviour support Plans |
| De Cuyper et al. 2021 | Flemish guideline for the prevention and use of seclusion and restraint. | Multidisciplinary guideline |
| Dekker-van Doorn et al. 2020 | Adaptive design: adaptation and adoption of patient safety practices in daily routines, a multi-site study. | Patient safety practices |
| Delaney et al. 2001 | Developing a Restraint-Reduction Program for Child/Adolescent Inpatient Treatment. | n/a |
| Dickens et al. 2020 | Safewards: Changes in conflict, containment, and violence prevention climate during implementation. | The Safewards |
| Dike et al. 2021 | Implementing a program to reduce restraint and seclusion utilization in a public-sector hospital: Clinical innovations, preliminary findings, and lessons learned. | Alternatives to Restraint and SeclusionState Incentive Grant (ARS-SIG) |
| Dols et al. 2010 | Terugdringen van separaties, waar stan we en hoe verder?: Een opinierende bijdrage. | A quality criteria for the use of seclusion and restraint |
| Donnelly et al. 2021 | Staff perceptions and implementation fidelity of an autism spectrum disorder care pathway a child/adolescent general psychiatric inpatient service. | Autism Spectrum Disorder Care Pathway |
| D’Orio et al. 2004 | Reduction of episodes of seclusion and restraint in a psychiatric emergency service. | A comprehensive plan focusing on early identification and management of problematic behaviors |
| Duxbury et al. 2019 | Minimising the use of physical restraint in acute mental health services: The outcome of a restraint reduction programme (‘REsTRAIN YOURSELF’). | REsTRAIN Yourself |
| Duxbury et al. 2019b | Staff experiences and understanding of the REsTRAIN Yourself initiative to minimize the use of physical restraint on mental health wards. | REsTRAIN Yourself |
| E-Morris et al. 2010 | Nurse-directed care model in a psychiatric hospital: a model for clinical accountability. | Nurse-Directed Care Model |
| Fagan et al. 2019 | Developing targeted MR-CRAS interventions to reduce the duration of mechanical restraint among forensic psychiatric inpatients | Mechanical Restraint – Confounder, Risk, Alliance Score (MR-CRAS) checklist |
| Fisher et al. 2003 | Elements of successful restraint and seclusion reduction programs and their application in a large, urban, state psychiatric hospital. | Restraint reduction program |
| Fletcher et al. 2020 | Safewards training in Victoria, Australia a descriptive analysis of two training methods and subsequent implementation. | The Safewards |
| Fletcher et al. 2017 | Outcomes of the Victorian Safewards trial in 13 wards: impact on seclusion rates and fidelity measurement. | The Safewards |
| Fluttert et al. 2010 | Preventing aggressive incidents and seclusion in forensic care by means of the ‘Early Recognition Method’. | Early Recognition Method |
| Foster et al. 1999 | Staff training decreases use of seclusion and restraint in an acute psychiatric hospital. | Staff training session on the management of assaultive behavior, weekly discussion items during team meetings for each local ward, and hospital-wide publicity charting the ongoing progress of the effort |
| Forsyth et al. 2018 | Sensory strategies in adult mental health: A qualitative exploration of staff perspectives following the introduction of a sensory room on a male adult acute ward. | Sensory room |
| Foxx et al. 1998 | A comprehensive treatment program for inpatient adolescents. | Behavioral program |
| Geller et al. 1992 | Clinical encounters with outpatient coercion at the CMHC: questions of implementation and efficacy. | Coerced community treatment |
| Geller et al. 2007 | Restraint and Seclusion: The Model for Eliminating Use in Health Care. | n/a |
| Geoffrion et al. 2018 | Impact of a program for the management of aggressive behaviors on seclusion and restraint use in two high-risk units of a mental health institute. | The Omega Program for the Management of Aggressive Behaviors |
| Georgieva et al. 2013 | Reducing seclusion through involuntary medication: a randomized clinical trial. | Involuntary medication |
| Giblin et al. 2012 | Reducing the use of seclusion for mental disorder in a prison: implementing a high support unit in a prison using participant action research. | High Support Unit |
| Gillies et al. 2005 | An innovation model for restraint use at the Philadelphia Veterans Affairs Medical Center. | Model for restraint use |
| Gonzalez-Torres et al. 2014 | Impact of the creation and implementation of a clinical management guideline for personality disorder in reducing use of mechanical restraints in a psychiatric inpatient unit. | A clinical management guideline for personality disorder |
| Goulet et al. 2018 | A case study: Seclusion and restraint in psychiatric care. | n/a |
| Goulet et al. 2018 | A pilot study of “post-seclusion and/or restraint review” intervention with patients and staff in a mental health setting. | Post-seclusion and/or restraint review |
| Gouzoulis-Mayfrank et al. 2019 | Öffnung einer psychiatrischen Akutstation als komplexe Intervention. | Open door policy |
| Griffith et al. 2021 | A clinical decision support system to prevent aggression and reduce restrictive practices in a forensic mental health service. | eDASA + APP |
| Guzman-Parra et al. 2016 | Effectiveness of a multimodal intervention program for restraint prevention in an acute Spanish psychiatric ward. | Regulatory Protocol for Mechanical Restraint and Coercion |
| Guzman-Parra et al. 2015 | Effects of a regulatory protocol for mechanical restraint and coercion in a Spanish psychiatric ward. | Multimodal intervention program based on six core strategies |
| Guzman-Parra et al. 2021 | A regional multicomponent intervention for mechanicalrestraint reduction in acute psychiatric wards. | Multicomponent intervention based on six core strategies |
| Haefner et al. 2021 | A Quality Improvement Project Using Verbal De-Escalation to Reduce Seclusion and Patient Aggression in an Inpatient Psychiatric Unit. | Verbal De-Escalation |
| Hambrick et al. 2018 | Restraint and critical incident reduction following introduction of the Neurosequential Model of the Therapeutics (NMT). | Neurosequential Model of Therapeutics |
| Hammer et al. 2016 | Effekten av komlementær ytre regulering (KYR) på tvangstiltak. | Complementary External Regulation |
| Hardesty et al. 2007 | Evaluation initiatives to reduce seclusion and restraint. | Multiple interventions focusing on trauma |
| Hellerstein et al 2007 | Decreasing the use of restraint and seclusion among psychiatric inpatients. | 1) decreasing initial time in restraint or seclusion from 4 to 2 hours before a new order was required; 2) education of staff concerning identification of patients at risk of restraint or seclusion and early interventions to avoid crises; and 3) use of a coping questionnaire to assess patient preferences for dealing with agitation. |
| Hochstrasser et al. 2018 | Long-term reduction of seclusion and forced medication on a hospital-wide level: implementation of an open-door policy over 6 years. | Open door policy |
| Holstead et al. 2010 | Restraint Reduction in Children's Residential Facilities: Implementation at Damar Services. | Multiple interventions |
| Hottinen et al. 2020 | Impact of the implementation of the Safewards model on the social climate on adolescent psychiatric wards. | The Safewards |
| Hube et al. 2021 | Restraint, seclusion and implementation of positive behavior interventions and supports. | Positive behavior interventions and supports |
| Huckshorn et al. 2004 | Reducing seclusion & restraint use in mental health setting: Core strategies for prevention. | Six core strategies |
| Huckshorn et al. 2005 | Six core strategies© to reduce the use of seclusion and restraint planning tool. | Six core strategies |
| Hyde et al. 2009 | A clinical improvement project to develop and implement a decision-making framework for the use of seclusion. | A clinical decision-making framework |
| Iennaco et al. 2017 | Aggressive Event Incidence using the Staff Observationof Aggression Scale-Revised (SOAS-R): A LongitudinalStudy. | n/a |
| Jackel et al. 2019 | Changes on ward atmosphere and job satisfaction after implementation of the Safewards model in two locked acute psychiatric wards - A multi-perspective evaluation. | The Safewards |
| Jani et al. 2011 | The implementation of milieu therapy training to reduce the frequency of restraints in residential treatment centers. | Milieu therapy training |
| Jensen et al. 1998 | Restraint reduction - A new philosophy for a new millennium. | Restraint reduction program |
| Jonikas et al. 2004 | A program to reduce use of physical restraint in psychiatric inpatient facilities. | Restraint reduction program |
| Jungfer et al. 2014 | Reduction of seclusion on a hospital-wide level: Successful implementation of a less restrictive policy. | Open ward treatment |
| Jäckel et al. 2019 | Changes on Ward Atmosphere and Job Satisfaction afterImplementation of the Safewards Model in Two Locked Acute PsychiatricWards – A Multi-Perspective Evaluation | The Safewards |
| Kazantsev et al. 2014 | Forensic psychiatric expertise and implementation of compulsory medical measures. | n/a |
| Ketelsen et al. 2007 | Kooperationsmodell zwischen psychiatrischen Kliniken mit dem Ziel der Qualitässicherung bei Zwangsmaßnahmen. | Cooperative Model |
| Kilgore et al. 2018 | Effectiveness of Collaborative Problem Solving Model in Reducing Seclusion and Restraint in a Child Psychiatric Unit. | Collaborative Problem Solving Model |
| Killick et al. 2005 | Training staff in an adolescent inpatient psychiatric unit in positive approaches to managing aggressive and harmful behaviour: Does it improve confidence and knowledge? | Positive Behaviour Management |
| Kleinmann et al. 2009 | Systems-level assessment of interobserver agreement (IOA) for implementation of protective holding (therapeutic restraint) in a behavioral healthcare setting. | n/a |
| Knox et al. 2007 | Reducing physical restraint use in residential aged care:implementation of an evidence-based approach toimprove practice. | Evidence-based approach |
| Kriegel et al. 2016 | Implementation and Outcomes of Forensic Housing FirstPrograms. | n/a |
| Lach et al. 2016 | Changing the Practice of Physical Restraint Use in Acute Care | n/a |
| Lang et al. 2017 | Introducing an open door policy in psychiatric inpatient units – practical implications and effects on coercive measures. | n/a |
| Lau et al. 2020 | Aims to reduce coercive measures in forensic inpatient treatment: A 9-year observational study. | Changes in the use of coercive measures and staff training |
| Lewis et al. 2009 | Crisis prevention management: A program to reduce the use of seclusion and restraint in an inpatient mental health setting. | An evidenced-based performance improvement program |
| Lickiewicz et al. 2021 | Reducing aggression in psychiatric wards using Safewards – A Polish study. | The Safewards |
| Long et al. 2015 | Reducing the use of secluision in a secure service for women. | Multiple interventions based on milieu therapy approach |
| Luiselli et al. 2008 | Effects of Fixed-Time Release (FTR) fading on implementation of physical restraint. | Fixed-Time Release |
| Lykins et al. 2016 | Implementing an Intervention to Reduce Seclusion and Restraints in an Inpatient Psychiatric Hospital. | Evidence-based program |
| Maguire et al. 2018 | Evaluating the introduction of the Safewards model to a medium- to long-term forensic mental health ward. | The Safewards |
| Mahler et al. 2021 | Weddinger Modell. Strenghtening the therapeutic relationship and preventing coercion in the treatment of persons with psychosis. | Weddinger Modell |
| Maitre 2018 | Psychiatric advance directives: Propositions for a specific model in France. | Psychiatric advance directives |
| Mann-Poll et al. 2018 | Long-term impact of tailored seclusion reduction program: Evidence for change? | Seclusion reduction program |
| Martin et al. 2008 | Reduction of restraint and seclusion through collaborative problem solving: A five-year prospective inpatient study. | Collaborative problem solving |
| Martin et al. 2004 | Seclusion and restraint “one-hour-rule”. | Legislative change called ‘One-Hour Rule’ |
| McCue et al. 2004 | Reducing restraint use in a public psychiatric inpatient service. | Six interventions primarily focusing on changing staff behaviour |
| McLoughlin et al. 2006 | The recovery model and seclusion and restraint. | n/a |
| Mekki et al. 2017 | The inter-play between facilitation and context in thepromoting action on research implementation in healthservices framework: A qualitative exploratory implementation study embedded in a cluster randomized controlled trial to reduce restraint in nursing homes. | n/a |
| van Melle et al. 2021 | Implementation of High and Intensive Care (HIC) in the Netherland: a process evaluation. | High and Intensive Care (HIC) |
| van Melle et al. 2020 | Does high and intensive care reduce coercion? Association of Hic Model fidelity to seclusion use in the Netherlands. | High and Intensive Care (HIC) |
| Mental Health Commission 2014 | Seclusion and restraint reduction strategy. | The Seclusion and Restraint Reduction strategy |
| Morrison et al. 2003 | An Evaluation of Four Programs for the Management of Aggression in Psychiatric Settings. | n/a |
| Needham et al 2004 | The effectiveness of two interventions in the management of patient violence in acute mental inpatient settings: Report on a pilot study. | The Brøset Violence Checklist and standardized training course in aggression management |
| Newman et al. 2018 | Effects of a staff training intervention on seclusion rates on an adult inpatient psychiatric unit. | Staff training intervention |
| Newton et al. 2020 | Implementation of a Violence Checklist to ReduceSeclusion/Restraint on Inpatient Psychiatry. | The Brøset Violence Checklist |
| Noorthoorn et al. 2016 | Seclusion reduction in Dutch mental health care: did hospitals meet goals? | n/a |
| Odgaard et al. 2018 | The impact of modified mania assessment scale (MAS-M) implementation on the use of mechanical restraint in psychiatric units. | Modified mania assessment scale (MAS-M) |
| Orlick et al. 2019 | Reducing use of physical and chemical restraints through enhanced de-escalation training in adult inpatient psychiatry. | De-escalation training |
| Paccione-Dyszlewski et al. 2012 | A crisis management quality improvement initiative in a children’s psychiatric hospital: design, implementation, and outcome. | Crisis management program |
| Page et al. 2021 | ‘Today We Talked’: using a collaborative approach to transform care in the psychiatric intensive care unit. | ‘Today We Talked’ approach |
| Pérez-Revuelta et al. 2021 | Factors associated with the use of mechanical restraint in a mental health hospitalization unit: 8-year retrospective analysis. | n/a |
| Piccoli et al. 2014 | Planning and implementation of an open door psychiatric ward: the assessment of an experience in Piedmont. | Open door policy |
| Pollastri et al. 2016 | Minimizing seclusion and restraint in youth residential and day treatment through site-wide implementation of Collaborative Problem Solving. | Collaborative Problem Solving |
| Pollmächer et al. 2019 | Autonomy focusing as guiding idea of minimally restrictivepsychiatry. | n/a |
| Port et al. 2021 | Medication and physical restraint utilization following implementation of a patient behavioral event response algorithm: A retrospective case-control analysis. | Patient behavioral events response algorithm |
| Prescott et al. 2007 | Reducing mechanical restraints in acute psychiatric cate setting using rapid response teams. | Rapid response teams |
| Price et al. 2016 | Evaluation of Safewards in forensic mental health. | The Safewards |
| Putkonen et al. 2013 | Cluster-randomized controlled trial of reducing seclusion and restraint in secured care of men with schizophrenia. | Six core strategies |
| Reynolds et al. 2019 | Implementation of Modified Positive Behavioral Interventions and Supports in a youth psychiatric partial hospital program. | Modified Positive Behavioral Interventions and Supports |
| Riahi et al. 2016 | Implementation of the six core strategies for restraint minimization in a specialized mental health organization. | Six core strategies |
| Riahi et al. 2014 | Implementation of recovery rounds in the prevention of restraint and seclusion. | Six core strategies |
| Sarver et al. 2019 | Implementation of the Broset Violence Checklist on an acute psychiatric unit. | Brøset Violence Checklist |
| Schön et al. 2018 | Psychiatric service staff perceptions of implementing a shared decision-making tool: a process evaluation study. | Shared decision-making tool |
| Seckman et al. 2017 | Evaluation of the use of a sensory room on an adolescent inpatient unit and its impact on restraint and seclusion prevention. | Sensory room |
| Shields et al. 2021 | Decline in physical restraint use following implementation of institutional guidelines. | Multiple interventions |
| Shields et al. 2020 | The effect of centers for medicate and medicaid’s inpatient psychiatric facility quality reporting program on the use of restraint and seclusion. | The Centers for Medicare and Medicaid’s Inpatient Psychiatric Facility Quality Reporting (IPFQR) |
| Singh et al. 2020 | Comparative Effectiveness of Caregiver Training in Mindfulness-Based Positive Behavior Support (MBPBS) and Positive Behavior Support (PBS) in a Randomized Controlled Trial. | Mindfulness-Based Positive Behavior Support (MBPBS) and Positive Behavior Support (PBS) |
| Sivak et al. 2012 | Implementation of comfort rooms to reduce seclusion, restraint use, and acting-put behaviors. | Comfort rooms |
| Smalls et al. 2004 | Utility of the implementation of programmatic systems to reduce and eliminate restraint use for the treatment of problem behaviors with individuals with mental retardation | Multiple interventions |
| Smidth et al. 2019 | Implementing de-escalation in emergency units in psychiatric and general hospitals in Slagelse – a pilot study | De-escalation |
| Smith et al. 2003 | Implementing a community group on an adolescent psychiatric intensive care unit. | Community group |
| Smith et al. 2005 | Pennsylvania state hospital system’s seclusion and restraint reduction program. | n/a |
| Steinert et al. 2009 | Quality of processes and results in psychiatry: Decreasing coercive interventions and violence among patients with personality disorders by implementation of a crisis intervention ward. | Crisis intervention |
| Stensgaard et al. 2018 | Implementation of the Safeward model to reduce the use of coercive measures in adult psychiatric inpatient units: An interrupted time-series analysis. | The Safewards |
| Sullivan et al. 2004 | Effects of patient-focused care on seclusion in a psychiatric intensive care unit. | Patient-focused nursing care |
| Teitelbaum et al. 2007 | Multisensory environmental intervention (Snoezelen) as a preventive alternative to seclusion and restraint in closed psychiatric wards. | Multisensory environmental intervention (Snoezelen) |
| Thomas et al. 2005 | The management of aggression care plans: Implementation and efficacy in a forensic learning disability service. | Management of aggression care plans (MOACAP) |
| Thornicroft et al. 2013 | Clinical outcomes of Joint Crisis Plans to reduce compulsory treatment for people with psychosis: a randomized controlled trial. | Joint Crisis Plans |
| Trauer et al. 2010 | Evaluation of the effect of a structured intervention for the management of behavioural disturbance on the level of seclusion in an acute psychiatric inpatient ward. | The Management of Acute ArousalProgramme (MAAP) |
| Velasquez et al. 2020 | Implementation of a specialized program to treat violence in a forensic population. | Enhanced TreatmentProgram (ETP) |
| Voskes 2015 | No effect without ethics: Reduction of seclusion in psychiatry from a care ethics perspective. | n/a |
| Werner et al. 1994 | Individualized care alternatives used in the process of removing physical restraints in the nursing home. | Individualized care alternatives |
| Whitecross et al 2020 | Implementing a psychiatric behaviours of concern team can reduce restrictive intervention use and improve safety in inpatient psychiatry. | Psychiatric behaviour of concern (Psy-BOC) team |
| Whitmore et al. 2017 | Evaluation of Safewards in forensic mental health: a response. | The Safewards |
| Wieman et al. 2014 | Multisite study of an evidence-based practice to reduce seclusion and restraint in psychiatric inpatient facilities. | Six core strategies |
| Winston et al. 1999 | Improving patient care through implementation of nurse-driven restraint protocols. | Nurse-driven restraint protocols |
| Wolf et al. 2021 | From Wish to Reality: Soteria in Regular Care—Proof of Effectiveness of the Implementation of Soteria Elements in Acute Psychiatry. | Soteria elements |
| Wullschleger et al. 2018 | Can “Model projects of need-adapted care” reduce involuntary hospital treatment and the use of coercive measures? | Model project of need-adapted care |
| Zetterberg et al. 2016 | Translating Coercion Policy into Inter-Organisational Collaboration–the Implementation of Compulsory Community Care for People with Mental Illness. | Compulsory Community Care |
| Zuehlke et al. 2016 | Brief report: Transformation to a Recovery-Oriented Model of Care on a veterans administration inpatient unit. | Recovery interventions |

n/a= no intervention implemented
